# Licit use of illicit drugs for treating depression: the pill and the process

**DOI:** 10.1172/JCI180217

**Published:** 2024-06-17

**Authors:** Alejandro Torrado Pacheco, Bita Moghaddam

**Affiliations:** 1 Department of Behavioral Neuroscience, and; 2 Department of Psychiatry, Oregon Health & Science University, Portland, Oregon, USA.

## Abstract

Psilocybin, MDMA, and ketamine have emerged as potentially effective treatments for rapid amelioration of the symptoms of mood and related psychiatric disorders. All clinical data collected so far with regard to psilocybin or MDMA, which have reported positive outcomes for treating depression, anxiety, posttraumatic stress disorder, and drug or alcohol use disorders, have involved clinician-assisted intervention. While the case for ketamine is assumed to be different, the first report of the successful use of ketamine in psychiatry for treating depression was in combination with psychotherapy, and an emerging literature suggests that the subjective state of individual experiences with ketamine predicts clinical outcome. This Review will focus on (a) a brief review of the literature, showing that the context or the process of drug administration has been an integrative component of published work; (b) the importance of clinical trials to compare the efficacy of the drug (“pill”) as a stand-alone treatment versus drug in combination with clinician-assisted psychological support (“process”); and (c) suggestions for future approaches in animal models that take into account the role of systems and behavioral neuroscience in explaining a potential role for context, experience, and expectancy in drug effect.

## Introduction

Three illicit drugs — psilocybin, 3,4-methylenedioxymethamphetamine (MDMA), and ketamine — have emerged as “novel” rapid-acting psychotherapeutic aids for mental health treatment. These compounds are not novel in that they have been around for decades and used and abused for recreational purposes. The novelty designation refers to their clinical effects, which cause the field to question much of our understanding of the mechanisms that guide pharmacotherapy of psychiatric disorders. First, the clinical efficacy of these drugs often emerges after 1 to 2 doses, and it tends to be rapid and long lasting (1–3). This is in contrast to most mainstream pharmacological approaches where either the effect emerges after several weeks of treatment — such as with classic antidepressants — or is seen transiently when the drug is on board — such as with benzodiazepine treatment of anxiety. Second, their reported clinical efficacy is transdiagnostic: at least, with two of these drugs, positive clinical outcomes are reported in multiple conditions, including depression, anxiety, posttraumatic stress disorder (PTSD), and substance use disorders (SUD) (1–4). Third, the clinical effect of these compounds may be impacted by the context in which they are received, by concurrent clinical interventions, such as psychotherapy, and by expectancy effects.

Use of these three compounds, which are often grouped under the umbrella term “psychedelics,” is receiving enormous attention, and various modes of treatment employing them are currently being tested. Our mechanistic understanding of how these drugs exert their clinical efficacy, however, remains scant. This is, in part, because many of the basic science approaches to study psychiatric drug effects were designed to address the mechanism of action of conventional antidepressant or anxiolytic treatments (5, 6). In the context of the therapeutic actions of psilocybin, MDMA, and ketamine, a critical mechanistic question is whether drug action alone, or drug interaction with the setting in which it is received, leads to positive effects on disease symptomatology. Here, we will outline the current state of the field, particularly as it relates to this issue, and identify future challenges in basic and clinical research for identifying the mechanism of action of these drugs.

## Beyond psychedelics: reclassifying MDMA, ketamine, and psilocybin

The term psychedelics traditionally refers to three classes of substances that include phenethylamines, tryptamines, and ergolines (7). Examining the subtle differences between these classes and their members is beyond the scope of this Review, but it is useful to keep in mind that a variety of compounds are included under the umbrella of psychedelic drugs. Classical psychedelics are generally grouped together on the basis of their principal pharmacological mechanism of action, which is to bind as full or partial agonists to the 2A subtype of the serotonin (5-HT) receptor (5HT2A) (7–9). These compounds, however, also bind to several other receptors, serotonergic subtypes, as well as other types (7, 8). The most notable and well-known members of the classical serotonergic psychedelics are lysergic acid diethylamide (LSD) and psilocybin, the active compound in several mushroom species with psychoactive effects.

MDMA and ketamine have profoundly different pharmacological mechanisms of action and subjective effects despite some attempts to call them psychedelics. MDMA has prosocial and euphoric effects, and, while it does influence the serotonergic system, it does so by promoting global serotonin release and potentially producing indiscriminate activation of all serotonin receptors (10), as opposed to the relatively selective activation of certain 5-HT receptors by classical psychedelics. Ketamine is an altogether different chemical, acting chiefly on the n-methyl-d-aspartate (NMDA) subtype of glutamate receptors and having a dissociative or anesthetic effect, depending on the dose (11). Thus, while these two compounds have in common with psilocybin the potential for treating symptoms of psychiatric disorders, here we will refrain from grouping these rapid-acting psychotherapeutics under the arbitrary label of psychedelics and instead focus on the specific features of each drug and the clinical data that support their use as therapeutic agents.

## Psychotherapeutic features of MDMA, psilocybin, and ketamine

### MDMA.

MDMA increases openness and prosocial behaviors, inducing feelings of trust and emotional connection in the user (12). These qualities naturally led to the idea that MDMA may enhance the effectiveness of behavioral therapy by creating a state of increased openness and trust, fortifying the alliance between therapist and patient (13, 14).

Because of these features and its effects on reducing fear, including evidence of enhanced fear extinction in laboratory animals (15–17), MDMA was proposed as a possible therapy aid for PTSD (18). Current treatment options for PTSD include psychotherapy as well as antidepressant and other forms of pharmacotherapy to treat specific symptoms (19, 20). A large proportion of patients, however, do not respond adequately to currently FDA-approved medications (18–20). Moreover, psychotherapy alone is ineffective for the treatment of PTSD in a substantial fraction of patients, and it is not uncommon for symptoms to persist for years or even decades (19).

Psychotherapy assisted with MDMA, however, may revolutionize treatment of PTSD. The first phase II pilot study showed promising results, with a substantial decrease in PTSD symptoms, as assessed using the gold-standard Clinician-Administered PTSD Scale (CAPS) up to the last measured time point, 2 months after dosing (21). This landmark study effectively set the blueprint for most other clinical trials using MDMA by establishing guidelines for the use of MDMA in combination with psychotherapy. These guidelines (22), inspired by early psychiatric work with MDMA and classical psychedelics (23), have been updated several times and are available via the Multidisciplinary Association for Psychedelic Studies (MAPS; recently rebranded to Lykos Therapeutics). Briefly, patients receive two nondrug psychotherapy sessions with a team of two therapists in the weeks before the experimental sessions to both prepare them for the experience and to establish a relationship with the therapists. The experimental sessions were conducted in the presence of the therapy team. Participants reclined on a futon or sofa, were provided with optional eye shades, and listened to music. The psychotherapy provided was nondirective, and patients were encouraged to alternate between discussion and introspection. The duration of the session was determined by the duration of the drug experience. Several other nondrug therapy sessions, known as integration sessions, were conducted thereafter.

This model, of minimal nondirective psychological support during the drug experience preceded by preparatory sessions and followed by integration sessions, has been used in most clinical trials of MDMA so far. A pooled analysis of six clinical trials compared control doses (placebo or 25 mg or 40 mg MDMA) with active doses (75 mg, 100 mg, or 125 mg MDMA). Five of these six trials used the psychotherapy model outlined above (24). Results showed a significant improvement in symptoms of PTSD after two psychotherapy sessions combined with a high dose of MDMA and a further improvement after a third session for participants who were offered and opted to receive it. The control groups also show improved CAPS scores, though the magnitude of this improvement was smaller. Notably, these improvements were shown to last well beyond the drug sessions, with low CAPS scores up to 12 months later in one study (25) and a follow-up study showing an enduring effect for 75%–90% of participants up to 7 years after the sessions (26). The effectiveness and safety of MDMA-assisted psychotherapy for PTSD has resulted in the approval of phase III clinical trials, the results of which were recently published (27, 28). These studies reported significantly reduced CAPS scores for participants receiving MDMA-assisted therapy compared with placebo with psychotherapy as well as increased incidences of loss of diagnosis and remission. Following these successful trials, MAPS Public Benefit Corporation (a clinical-stage company) announced the submission of a new drug application to the FDA for MDMA used in combination with psychological intervention.

### Psilocybin.

While MDMA was not used formally in therapeutic settings until the 1970s, the use of classical psychedelics in research settings dates back to the years between 1943 and 1970, i.e., between the discovery of the subjective effects of LSD and the passing of the Controlled Substances Act by the Congress of the United States (29, 30). Several clinical studies were conducted in this period suggesting that psychedelics could be very powerful as a therapeutic aid. In contrast to the later work with MDMA, the therapeutic approach used in this work is often not well described. Although these trials are not up to par with modern standards, encouraging results were commonly reported (29, 30). These early studies primarily employed LSD as the drug of choice, as it was more readily available than psilocybin. Modern day psychedelic research has instead mainly employed psilocybin, which has quickly become a candidate drug for the treatment of several psychiatric disorders, when used in combination with psychological intervention.

Clinical trials with psilocybin in the 20th century have largely focused on anxiety and depression. Improvements in symptoms have been reported in patients with life-threatening cancer (31–33), and an open-label trial of psilocybin for treatment-resistant depression showed a significant reduction in the severity of symptoms following psilocybin sessions (34). Recent trials have replicated these findings in larger patient samples and increased our understanding of the parameters that result in improvement of symptoms (35–38). First, the reduction in depression scores depends on the dose of psilocybin, with only high doses conferring therapeutic benefits (37). Second, the amelioration of symptoms is long-lasting, with effects maintained for several months and as long as one year after the psilocybin sessions (39). Finally, two psilocybin sessions, three weeks apart, improved depression scores over a six-week time period to a similar extent as daily repeated escitalopram, a selective serotonin reuptake inhibitor (SSRI) that is an established treatment for depression (40).

Psilocybin has also shown promise as a potential treatment for SUD and alcohol use disorders (AUD). This is once again in line with historical research with LSD, which was tested as a treatment for AUD in the mid-twentieth century (41). An initial pilot study with psilocybin showed encouraging results for patients with AUD (42). A later randomized, double-blind, placebo-controlled trial showed a reduction in drinking and heavy drinking in the psilocybin group that was significantly greater than that in the placebo group (43). Another study examined the use of psilocybin in combination with psychotherapy for smoking cessation. With the caveat that this was a pilot study with a small number of participants and an open-label design, abstinence at 6 months and 12 months was maintained in 60% of participants, a percentage that is much higher than that achieved with current treatments (44, 45).

In the psilocybin trials for depression summarized above, the design used is relatively similar to the one pioneered in the MDMA-assisted treatment of PTSD. The support provided is nondirective, and participants are encouraged to focus on introspection. Interestingly, clinical trials studying psilocybin in the context of SUD have not followed this model, opting instead to use cognitive behavioral therapy during the psilocybin sessions (42–45). This may underscore the fact that the currently used standard for psilocybin-assisted psychotherapy has largely been adopted from the work done with MDMA research. It is interesting, however, that the subjective effects of these two drugs are substantially different. With MDMA, the prosocial effects provide a clear interpretive framework to explain why the therapeutic alliance is strengthened. Similarly, its reduction of fear responses logically leads to its use in treating PTSD, where accessing and reprocessing traumatic memories is the accepted mode of treatment. In contrast, no such clear or direct connection between psilocybin’s subjective effects and its apparent therapeutic benefits has been put forward.

### Ketamine.

Using ketamine to treat depression and symptoms of other (nonpsychotic) psychiatric illnesses was first reported in 1973 (46). Two physicians at the Pahlavi University in Shiraz used a low dose of ketamine in conjunction with psychotherapy, similar to the MDMA studies described above, in 100 patients who were hospitalized for a variety of psychiatric symptoms. They observed lasting reduction in mood- and anxiety-related symptoms in the majority of patients after six-month and one-year follow-ups.

This research did not get much attention, perhaps because at that time the preferred way of treating mood disorders was rapidly shifting away from psychotherapy to the use of antidepressant drugs. Coincidentally, the first report of the discovery of antidepressant drug fluoxetine (Prozac) was in 1974 (47). Fast forward to 2000, when a report showed that a single low dose of ketamine can attenuate symptoms of depression in treatment-resistant patients (48). This study was in only seven patients, and it lacked a placebo control, but it was remarkable nonetheless because it questioned the accepted assumption that antidepressants take several weeks to work. This limited study was followed by several large-scale and well-controlled studies in patients with treatment-resistant depression (TRD). These studies showed modest but significant effects of ketamine on symptoms of TRD (49). This was an exciting finding because while a majority of patients respond to first-line treatments for depression (i.e., SSRIs or serotonin and noradrenaline reuptake inhibitors), about 30% present with TRD. Subsequent studies (50, 51) using intranasal application of one of ketamine’s enantiomers, esketamine (Spravato), were promising enough to convince the FDA in United States and the European Commission to approve the use of ketamine for treatment of depression in 2019.

More recent clinical trials have shown a range of strong to weak to no significant effects in relieving symptoms of depression (49, 52, 53, 54). Unlike MDMA and psilocybin, ketamine is readily available. This has led to booming business with hundreds of clinics and spas that provide ketamine infusions to treat depression. There are concerns about its unregulated use with vulnerable individuals paying a lot of money for repeated ketamine infusions when, in fact, we have limited data on how repeated use of ketamine can influence the function of the human brain. The clinical long-term efficacy of ketamine appears to be highly variable (54). Moreover, repeated exposure to ketamine can lead to adverse and dangerous peripheral side effects, such as ulcerative cystitis and the so-called ketamine bladder syndrome (55). The need to repeatedly administer ketamine to maintain antidepressant effects, or its limited or lack of clinical efficacy in many individuals, is hardly mentioned in media stories about the drug.

Despite recent safety concerns and low effect size in published clinical trials, ketamine appears to have impressive antidepressant effects in some individuals with TRD. What explains the variability in its clinical efficacy? Clues can come from the original 1973 study that showed a sustained success rate when one or two low doses of ketamine were given to an in-patient population in conjunction with psychotherapy. This suggests that there may be a critical relationship between context and subjective drug experience and the therapeutic efficacy of ketamine. This is supported by recent reports that have shown a relationship between individuals’ subjective and emotional reactions to ketamine infusion and how well the symptoms of depression are treated, though this effect is not consistent across studies (56). Notably, data from patients receiving ketamine-assisted therapy shows benefits that may potentially outperform ketamine alone (57). The antidepressant effectiveness of ketamine may, therefore, be related to the emotional and affective state of individuals at the time that they were receiving the drug. These findings clearly need to be replicated and several ongoing clinical trials combining ketamine with psychotherapy in TRD are indeed ongoing. A recent study demonstrating that the antidepressant effects of ketamine administered under anesthesia correlated better with the assumption of having received ketamine (versus actually receiving ketamine) further supports the idea that context and setting — in this case expectancy — may contribute to ketamine’s therapeutic efficacy (58).

## Common neurobiological mechanisms for rapid-acting psychotherapeutics

**Figure 1:**
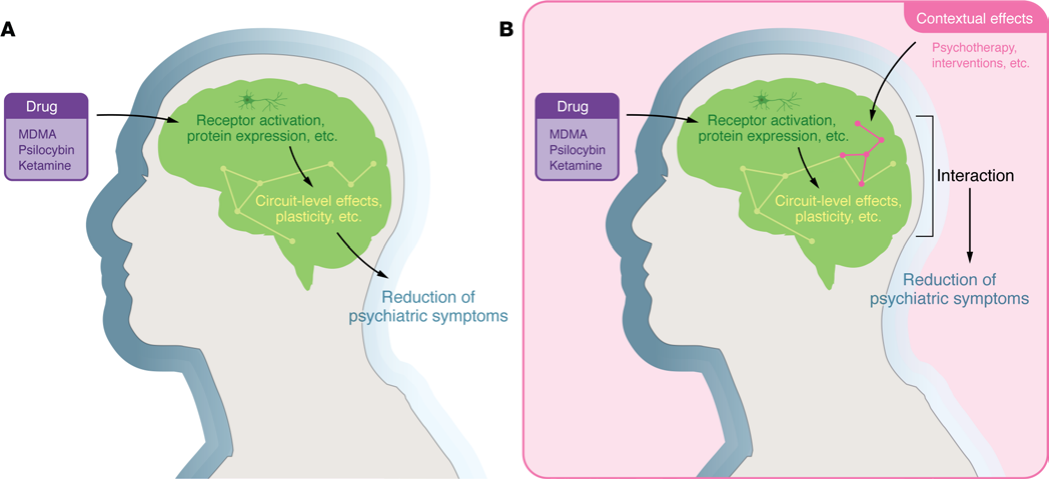
Two potential paths for the mechanism of action of rapid-acting psychotherapeutics: the pill and the process. (**A**) In the “pill” model, ketamine, MDMA, and psilocybin produce their clinical efficacy by acting purely as pharmacological agents. In this view, their actions on specific receptors and individual neurons influence isolated brain networks that directly lead to behavioral changes and alleviation of symptoms. (**B**) In “the pill and the process” model, an interaction between the brain state and drug effect leads to engagement of new brain networks that lead to alleviation of symptoms in a context-dependent manner. Thus, the receptor activity and other neurophysiological effects of these drugs produce an interactive network state that changes the ongoing and future computation of context, memory, or subjective states (such as states brought upon by concurrent psychological intervention). This interactive state will then result in a different mode of network engagement than the drug alone. In this model, positive effect on symptoms of psychiatric illness will, therefore, depend on the effectiveness of the coinciding intervention.

There are two fundamentally distinct approaches to address whether rapid-acting psychotherapeutics share common mechanisms (Figure 1). The first is that the therapeutic benefits of MDMA, psilocybin, or ketamine are due to the drug action alone (“the pill”). The second is that these psychoactive drugs modify how context and internalized information, including memories, are encoded and processed by the brain (“the process”). While we have made excellent progress in establishing drug effect alone at molecular or cellular level, there is a glaring lack of information about the systems and behavioral neuroscience of these drugs when they are administered under different settings and experiences. We explore these two questions separately below.

### In support of the process.

While with MDMA, the literature clearly supports therapeutic efficacy when the drug is combined with psychotherapy, it is generally assumed that psilocybin or ketamine may work as standalone pharmacological agents. Ketamine has been used without clinician-assisted therapy or controlling context in multiple clinical trials. While that may explain its widely variable results, the question of whether it alone can more effectively alleviate depression if the context is controlled remains open for testing (59). A recent study in which individuals with TRD were given ketamine blindly while they were anesthetized reported similar efficacy between ketamine and placebo, suggesting that expectancy or context play a role in the reported therapeutic effects of ketamine, as opposed to drug effect alone (58). With psilocybin, the literature reviewed above makes it evident that all clinical trials to date have used a combination of psychotherapy or other forms of clinician-assisted psychological support. Moreover, it is well established that psilocybin can cause deeply meaningful experiences, often described as “peak” or “mystical” experiences (60). There is mounting evidence that the occurrence and strength of these experiences is correlated with changes in depression scores (61). This observation has led to the hypothesis that the subjective experience is causally related to the therapeutic outcome. The data available, however, are not definitive, as many plausible tests of this theory, such as administration of psilocybin (or MDMA) during anesthesia or in conjunction with memory-impairing drugs, such as midazolam (62), have not been performed.

### In support of the pill.

It has been suggested that dissociative effects of ketamine or mystical experiences of psilocybin may simply be an epiphenomenon and that they may not be required for the therapeutic effects of these drugs (63). If this were the case with psilocybin, 5HT2A-selective agonists that do not cause these subjective effects could be a promising therapeutic target. Whether this hypothetical drug could be as effective as psilocybin in reducing symptoms of depression or anxiety will only be determined by appropriate clinical trials. Regardless, even if such a compound were available, the question of whether it would be clinically effective without concurrent psychotherapy remains.

The idea that the subjective experience may be superfluous is based on the observation that fast-acting antidepressants (including psilocybin and ketamine) may share the ability to rapidly induce neuronal plasticity in the cerebral cortex (64, 65) and other forebrain regions (Table 1). Psilocybin and other 5H2A agonists (66) produce structural and synaptic plasticity in the frontal cortex and hippocampus of mice (67–69) similar to ketamine. It is, therefore, suggested that these forms of plasticity are a common mechanism that cause therapeutic benefits of psilocybin, ketamine, and other fast-acting antidepressants (70).

While this hypothesis is attractive, it should be underscored that there is no clear link between the observed effect of promoting plasticity and amelioration of depressive symptoms. In fact, increased plasticity may not always be a good thing: many other drugs produce similar patterns of plasticity in rodents, including, for instance, repeated exposure to amphetamine and cocaine (71, 72). In fact, drug-induced structural plasticity in the prefrontal cortex was first described in 1997 in response to repeated amphetamine exposure (73, 74). Moreover, a recent study has questioned the methodological validity of reports of psychedelic-induced neuroplasticity (75). Nevertheless, it is intriguing to hypothesize that rapid neuroplasticity induced by ketamine or psilocybin produces therapeutic effects by virtue of modifying how affective or cognitive events are processed. One possibility is that increased plasticity is related to enhancements in cognitive flexibility that have been observed in humans and rodents upon psilocybin administration (76, 77). Alternatively, it could be a question of reopening a critical period of plasticity relevant to traumatic memories, emotional processing, or development of the self (78). Importantly, if the latter is a relevant mechanism at work, psychotherapy would be an essential part of treatments involving these drugs, which would focus on leveraging the impact of increased plasticity toward a positive clinical outcome (79).

This leads to another point for consideration: the therapeutic approach used so far in all clinical trials of psilocybin may not be the most effective. Is it possible that an approach more tailored to leverage the subjective effects of psychedelics could produce even greater benefits for depressed patients? Interestingly, some ongoing trials for SUDs are employing therapeutic approaches with well-established efficacy for the disorder in question, namely cognitive behavioral therapy (NCT05452772) or motivational enhancement therapy (NCT06225232, NCT05995769). The approach to use for depression and anxiety is less clear. Several proposals have been made that align with the ideas above, i.e., to tailor the therapeutic approach to the cognitive and subjective effects of the drug (79, 80). For psilocybin, this may mean an approach centered on behavioral flexibility (81) or focusing on interpersonal dynamics (80). An important element of future clinical trials of psilocybin-assisted psychotherapy should be to incorporate comparisons of different therapeutic approaches in the trial design, in addition to comparisons of psilocybin alone versus in conjunction with specific psychotherapeutic approaches.

## Moving forward: employing behavioral and systems neuroscience approaches

As mentioned above, there are comparatively more studies looking at the molecular and cellular effects of rapid-acting psychotherapeutics than examining their effects on clinically relevant behaviors and neural circuitry, particularly in preclinical model organisms. There is a glaring lack of information about the systems and behavioral neuroscience of these compounds and particularly of psychedelics.

Ketamine and psilocybin have in common the ability to induce rapid plasticity (Table 1). Ketamine mediates at least some of its effects on rodents via the tropomyosin kinase receptor B/brain-derived neurotrophic factor (TrkB/BDNF) pathway. In the hippocampus, TrkB and BDNF are required for ketamine-dependent synaptic potentiation, which correlates with antidepressant-like effects in mice (82). Interestingly, both traditional antidepressants (SSRIs, such as fluoxetine) and ketamine bind directly to TrkB (83). Recently, a study suggested that plasticity induction via activation of the TrkB/BDNF pathway may generalize to LSD and psilocybin (84), providing a potential convergent pathway for novel and traditional antidepressants, though this study awaits replication.

While these data inform us about the molecular pathways modulated by these psychoactive drugs, our understanding of the functional implications at the levels of brain networks and behavior is very limited. Studies investigating the physiological effects of psychedelics have begun to shed light on their effects on neuronal activity and neurotransmitter release, particularly in frontal cortex and some subcortical areas (85, 86). One issue is that a variety of compounds have been used in these studies, such as LSD, 5-MeO-DMT, 2,5-dimethoxy-4-iodoamphetamine (DOI), psilocybin, and others. However, these drugs all have slightly different activities at a multitude of receptors, which raises issues regarding how comparable their effects are when applied at a systemic, brain-wide level.

We suggest, as others have (85), that the field is in need of studies using in vivo neural recording methods in behaving rodents to further our understanding of the network-level effects of psilocybin and other psychedelics. Systems-level approaches are needed to understand how the complex pharmacology of psychedelic drugs works to modulate neural network activity and, in turn, how this produces the observed behavioral effects. In behaving animals, neurons are not simply agents of linear signal propagation. Networks of neurons select, modulate, compute, and then generate adapted and specialized outputs — that are not a copy of the input — in a context- and cell- specific manner. Thus, controlling the “input,” as in stimulation of 5-HT2A receptor or inhibition of NMDA receptors, may not be sufficient to explain the systems and behavioral effects of these compounds. It is, therefore, critical that we investigate the activity of groups of neurons and how their collective responses (e.g., synchronization, periodicity) are dynamically influenced by these drugs in behaviorally active contexts.

Additionally, while theoretical ideas about how these drugs work are needed and interesting, they should be accompanied by neuronal data. In fact, gaining an understanding of network-level effects will refine our theoretical understanding of psychedelic action. For instance, a popular idea is that psychedelics that act as agonists at 5HT2A receptors increase brain entropy by relaxing the reliance on prior beliefs and tipping the balance toward external inputs (87). However, a specific test of this hypothesis in rodents found the opposite (88), with the psychedelic DOI resulting in reduced bottom-up sensory drive. This test may not be definitive, and it is certainly possible that different results would be obtained with other drugs or in other brain regions. But the point remains that it is essential to formally and rigorously test these hypotheses and formulate evidence-based ideas on the action of psychedelic drugs.

Finally, and most critically, there is a dearth of behavioral data with psychedelics in preclinical models. Moving forward, it is important to focus on novel behavioral approaches that have strong translational relevance and investigate the neural consequences of context or setting to acute and long-term drug effects. Ketamine and psilocybin have so far been primarily tested in traditional rodent tasks that had been used for classical antidepressant screening. These tasks are useful to characterize learned helplessness and behavioral despair, as measured by the tail suspension test and forced swim test (though there are caveats about the latter, see ref. 89), as well as antianhedonic properties that interestingly for psilocybin appear to be independent of 5HT2A receptor activation (69). Thus, there is a need for out-of-the box thinking, as these drugs may act by entirely different pathways than classical antidepressants and crucially by influencing behavior in a context-dependent manner. For instance, the evidence in humans and rodents showing an enhancement in flexibility with psilocybin (76, 77) may be translationally relevant if the interaction between this cognitive change and concurrent intervention is the key to the therapeutic effect. Another example is investigating contextual learning and “unlearning” associated with both aversive and rewarding outcomes, which may tap into memory mechanisms that are potentially modulated by these drugs.

## Conclusion

Psilocybin, MDMA, and ketamine have the potential of improving the quality of life in individuals with mood and addictive disorders and whose symptoms have not responded positively to conventional drug therapy. They also offer exciting new possibilities for enhancing our mechanistic understanding of the biological basis of these symptoms, because they appear to be working on entirely different cellular targets and brain pathways compared with most conventional pharmacological modes of treatment. Previous approaches of giving animals the therapeutic drug (such as classic antidepressants) and then observing what receptors or other proteins or neurons are affected in isolated systems has led to many debunked theories (such as the serotonin hypothesis of depression) and lack of progress in discovering novel therapeutics. Moving forward, applying older behavioral approaches used in studying classical antidepressants to these drugs may not be transformative because this will not address how these drugs are working in conjunction with psychotherapy, context, or expectancy. Thus, we believe the field will benefit from focusing on the study of how brain networks engaged in high-level behaviors (e.g., memory recall, executive function, social behaviors) are modulated by these drugs.

Finally, we highlight as a note of caution that psilocybin, MDMA, and ketamine are all illicit drugs, which have abuse potential. Their misuse can be dangerous if not deadly. The problem in advancing these drugs as therapies stems, in part, from the fact that they are considered “recreational” drugs. The hype that they “cure” depression or PTSD may add to the glamour of their use and give the false impression that, just like self-medicating physical pain with aspirin or acetaminophen, one can cure psychiatric symptoms simply by taking them as needed. Moreover, the difficulty in controlling compound purity and dosage can add to the dangers of the self-medication approach, especially as higher doses of any of these three drugs can produce entirely different and often deleterious effects. The clinical data reviewed here indicates that, in the right setting and under appropriate clinical care, these drugs can relieve symptoms in some individuals who have not responded to conventional treatments. It is, therefore, imperative that information about how these drugs work is clear and precise and that their recommended use remains cautious and evidence based, so that they remain available and safe for those who need them.

## Figures and Tables

**Table 1:**
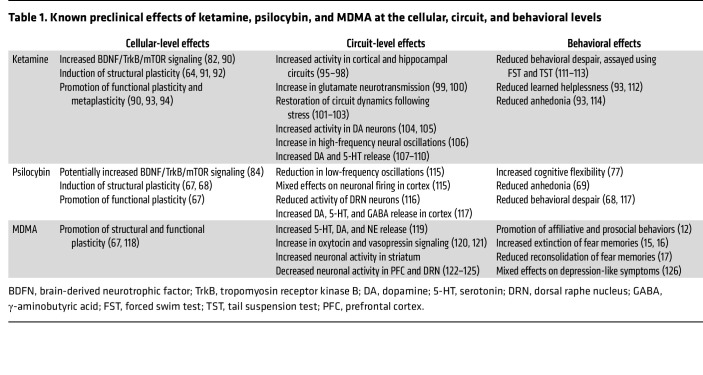
Known preclinical effects of ketamine, psilocybin, and MDMA at the cellular, circuit, and behavioral levels

## Version 1 06/17/2024

Electronic publication
